# Becoming popular: interpersonal emotion regulation predicts relationship formation in real life social networks

**DOI:** 10.3389/fpsyg.2015.01452

**Published:** 2015-09-29

**Authors:** Karen Niven, David Garcia, Ilmo van der Löwe, David Holman, Warren Mansell

**Affiliations:** ^1^Manchester Business School, University of ManchesterManchester, UK; ^2^Chair of Systems Design, ETH ZürichZürich, Switzerland; ^3^User Experience Research, Google, Inc., Mountain ViewCA, USA; ^4^School of Psychological Sciences, University of ManchesterManchester, UK

**Keywords:** interpersonal emotion regulation, emotion regulation, social networks, centrality, popularity, agreeableness, Twitter

## Abstract

Building relationships is crucial for satisfaction and success, especially when entering new social contexts. In the present paper, we investigate whether attempting to improve others’ feelings helps people to make connections in new networks. In Study 1, a social network study following new networks of people for a 12-week period indicated that use of interpersonal emotion regulation (IER) strategies predicted growth in popularity, as indicated by other network members’ reports of spending time with the person, in work and non-work interactions. In Study 2, linguistic analysis of the tweets from over 8000 Twitter users from formation of their accounts revealed that use of IER predicted greater popularity in terms of the number of followers gained. However, not all types of IER had positive effects. Behavioral IER strategies (which use behavior to reassure or comfort in order to regulate affect) were associated with greater popularity, while cognitive strategies (which change a person’s thoughts about his or her situation or feelings in order to regulate affect) were negatively associated with popularity. Our findings have implications for our understanding of how new relationships are formed, highlighting the important the role played by intentional emotion regulatory processes.

## Introduction

When we enter a new social situation, be it starting a new job, beginning a new course of study, moving to a new neighborhood, or even joining an online social network, forming connections with others is paramount to our satisfaction and success. But how can we develop these connections? An emerging body of research provides evidence that attempting to improve other people’s feelings may boost the quality of existing relationships (Niven et al., 2012a). The aim of the present paper is to investigate whether engaging in this process of interpersonal emotion regulation (IER) can help people to build *new* relationships during socialization in face-to-face and online networks.

The need to form high-quality relationships with others around us is considered to be a fundamental human motivation (Baumeister and Leary, 1995). Relationships with others can furnish people with many benefits, including practical and emotional support (Argyle, 1992). Perhaps unsurprisingly then, research in the social network tradition documents various advantages to being popular.

Popularity is typically defined as being well-liked and accepted by one’s peer group (Scott and Judge, 2009), and is, therefore, commonly operationalized as having a high number of connections to others in one’s social environment (Freeman, 1979). For example, many social network studies examine popularity by measuring *in-degree centrality*, which reflects the extent to which other people report having a connection with a focal person (Czarna et al., 2014). In work organizations, high popularity, as captured by a person’s in-degree centrality, is linked to better in-role and extra-role performance, higher well-being, and indicators of career success such as reputation and supervisors’ ratings of developmental potential (Sparrowe et al., 2001; Totterdell et al., 2004; Lin and Huang, 2005; Mehra et al., 2006). During socialization in new networks in particular, building informal connections with others can be crucial. In a study of newcomers to an accounting firm, for example, Morrison (2002) reported that the number of connections newcomers had formed during their first 9 months in post-predicted their social integration, learning, and commitment to the organization.

Connecting with others is not just important in face-to-face contexts, but also online. Over the past 10 or so years, use of social networking sites that allow people to establish and maintain connections with others online, such as Facebook and Twitter, has been growing at an incredible rate. In March 2012, such websites attracted audiences of almost 171 million unique visitors via computers and 67 million via mobile phones within France, Germany, Italy, Spain, and the UK (ComScore, 2012). Crucially, making connections on such sites is found to provide similar benefits as making connections in other oﬄine contexts. For example, Ellison et al. (2007) and Steinfield et al. (2008, 2009) have reported that connections through Facebook and internal organizational social networking sites are associated with greater well-being, social integration, and self-esteem.

Given the importance of forming new relationships for satisfaction and success, researchers have begun to explore factors associated with popularity. Typically, popularity has been examined as a function of observable attributes like gender at the neglect of psychological factors (Totterdell et al., 2008). Where psychological factors have been studied, the focus has usually been on stable traits (e.g., extraversion and agreeableness; Klein et al., 2004; Selfhout et al., 2010; Quercia et al., 2012), or similarity in demographic or personality characteristics (McPherson et al., 2001). Thus, to date, research has concentrated on predictors of popularity that people are largely unable to control.

In this paper, we introduce IER as a process that is under volitional control, which we argue might prove a fruitful avenue for investigation with respect to providing guidance about how to boost one’s popularity when entering new social contexts. We follow the definition offered by Niven et al. (2009) of IER as the process whereby people intentionally try to influence the way others feel. While others (Zaki and Williams, 2013) have used the term IER more widely, to refer to any form emotion regulation that involves more than one person, our use of the term specifically concerns attempts to regulate others’ feelings, rather than attempts to regulate one’s own emotions (in line with what Zaki and Williams term ‘extrinsic’ IER). Taking an example, if you just started work at a company and encountered a new coworker who appeared to be upset, you might offer to make a coffee for the person or make a light joke to try to cheer him or her up. If the same person appeared to be anxious, you might ask if he or she was okay and whether you could do anything to help. IER is used in a range of social contexts, including peer groups, support groups, sports teams, and work organizations (Thoits, 1996; Lively, 2000; Niven et al., 2012b; Friesen et al., 2013). Here, we maintain that IER might play an important role in building new relationships in such contexts.

The reason why IER is expected to play a role in forming new relationships is due to its link with positive affect. IER is most commonly used with the intention of improving others’ feelings, and evidence suggests that attempts to improve others’ feelings do often boost the intended target’s affect (Niven et al., 2007, 2012c), although the effects of offering support more generally may vary according to factors such as responsiveness to targets’ needs (Maisel and Gable, 2009). The often positive effects of attempts to improve others’ feelings likely transpire due to the social information communicated during IER (Van Kleef, 2009), as attempts to improve others’ feelings may convey positive information to the target (e.g., this person likes me and wants me to feel better). Not only do targets of IER experience changes to their affect, so too do those who observe IER (Totterdell et al., 2012). For example, studies of elevation describe the warm or glowing feeling that people experience when they witness acts of kindness or compassion toward others (Haidt, 2002). The effects of IER on observers’ affect are also likely to be due to positive inferences, this time on the part of the observer (e.g., about the agent’s motives and character, or about humanity more generally). Crucially, both targets and observers of IER attempts to improve others’ feelings are likely to attribute any pleasant emotion that results from this kind of interaction to the person who initiated the IER attempt.

The positive affect that may be arise from IER attempts could help to build new relationships in two ways. First, according to Lawler’s (2001) affect exchange theory, when pleasant feelings are experienced during an interaction, they trigger cognitive efforts to understand the causes (i.e., an attribution process; Weiner, 1986). Because people strive to reproduce pleasant feelings which are internally rewarding, if an exchange between *person a* and *person b* generates pleasant emotion which *person a* attributes to *person b*, *person a* will want to interact with *person b* again in the future, eventually generating a strong and durable network tie. Second, people may be drawn to others who leave them feeling positive because this enables them to conserve the cognitive resources that are typically associated with engaging in self-regulation of emotion. It is well-established that regulating one’s own emotions can be effortful and costly (Niven et al., 2013). Consistent with social baseline theory (Beckes and Coan, 2011) and Fitzsimons and Finkel’s (2010) notion of a shared regulatory system for emotions, building relationships with people whose IER is effective and results in pleasant feelings for the target may help to reduce those costs and may thus make an attractive proposition. As such, engaging in IER may help people to build relationships in newly formed social networks. However, to date, just two studies have reported a link between the use of IER and high-quality relationships, and the focus of those studies was on improving the quality of existing social ties (Niven et al., 2012a, Study 1 and Study 2) rather than on building new relationships.

Alongside the paucity of research regarding the potential role of IER in forming new connections stands the question of which types of IER are most important for building relationships. Building on work in the field of emotion self-regulation, which has distinguished between regulation that involves cognitive vs. behavioral means (Parkinson and Totterdell, 1999), as well research into the strategies that people use to regulate others’ feelings, the dominant model of IER proposes that strategies to improve others’ emotions can primarily be differentiated according to whether they are cognitive or behavioral (Niven et al., 2009, 2011). In IER terms, cognitive strategies involve trying to change a person’s emotions primarily by influencing the person’s thoughts about his or her feelings or situation (e.g., giving someone advice), while behavioral strategies involve trying to change a person’s emotions primarily by using one’s behavior to communicate a message about one’s relationship with the target (e.g., doing something nice for someone). Most studies to date on the effects of IER have yet to distinguish these strategy types. Here, we contend that these strategies may have different implications for the formation of new relationships, because of likely differences in how they are appraised by targets.

Cognitive strategies attempt to improve the target’s emotion by offering a different perspective, showing the situation in a different (usually positive) light. When it comes to regulating one’s own feelings, cognitive strategies, such as reappraisal, are usually considered highly effective (Gross and John, 2003; Webb et al., 2012). For regulating others’ emotions, however, such strategies could be seen by the target and by observers as a challenge to the target’s existing views. A key difference is that the change to the target’s view of the situation occurs by choice in the case of emotion self-regulation, but is enforced—and may not always be welcomed—in the case of IER. Thus, in the short-term at least, cognitive IER has the potential to be interpreted in negative terms, especially in a relationship that is not well established. Accordingly, cognitive IER may not always lead to a positive appraisal of the regulator’s motives and so may not make the target want to interact with the regulator in future.

Behavioral strategies, by way of contrast, attempt to change the target’s emotion by conveying a positive message about the agent’s relationship with the target that functions to express a sense of understanding and sharing of the target’s way of viewing the situation. Receipt of support, comfort, and validation are the motives most commonly-cited by people when they share negative emotions with others (Rimé, 2009). Thus, the use of such strategies in a new relationship would be likely to fulfill (and to be seen by observers to fulfill) the target’s needs, leading to a likely positive appraisal of the regulator’s intentions, and thus a positive relational outcome.

The evidence outlined above suggests that behavioral IER strategies would facilitate the development of new connections with others, whereas cognitive strategies may not always have the same benefits. In line with this proposition, a recent study in which pairs of friends or intimates were instructed to adopt specific listening strategies when discussing an emotional video sequence indicated that socio-affective strategies (which the authors likened to Niven et al.’s, 2009 behavioral strategies), but not reframing strategies (likened to cognitive strategies), led to feelings of emotional proximity and reduced loneliness (Nils and Rimé, 2012). However, to date, no studies have investigated whether spontaneous use of these distinct strategy types in everyday life has a differential impact on people’s relationship formation.

The studies presented in this paper present the first test of whether IER can help people to form new relationships, tracking the effects of IER on development of new connections in real social networks from the formation of networks over time. In Study 1, we test the effects of IER in face-to-face social networks. In Study 2, we build on our first study by contrasting the effects of cognitive and behavioral IER strategies, and by exploring the effects of IER in online social networks. Although traditionally it was assumed that the type of computer-mediated communication (CMC) that occurs online was devoid of social cues and, therefore, lacked emotional content, several perspectives challenge this view. For example, Walther’s (1992) social information processing theory argues that communicators are driven to develop social relationships, irrespective of their communication medium, and that relationships can, therefore, develop to the same degree via CMC as face-to-face communication. Recent accounts of emotion regulation further highlight that given that online exchanges may be just as emotional as face-to-face interactions, they should be included in contemporary studies of emotion in social contexts (Kappas, 2013).

## Study 1

In our first study, we examined whether IER could help people to form new relationships in face-to-face social networks. In particular, we investigated students taking year-long Masters courses, tracking the change in their popularity from the first few weeks of the course to the end of their first semester, and assessing their use of IER toward their coursemates in the interim period. In addition to assessing participants’ use of IER, we also measured two stable personality traits that have been found by previous researchers to be important predictors of popularity in social networks, namely extraversion and agreeableness (Selfhout et al., 2010; Quercia et al., 2012). Extraversion reflects individual differences in the extent to which people are outgoing, sociable, assertive, enthusiastic, and energetic, and thus may predispose people toward seeking out new relationships with others (Pollet et al., 2011). Agreeableness is a personality trait that reflects individual differences in sympathy, warmth, and consideration, and is strongly associated with motives to form positive relationships (Jensen-Campbell and Graziano, 2001).

We chose to examine two types of relationships in this context: work-related and non-work-related. In new organizational contexts, both of these relationship types are extremely salient and crucial for people to integrate into their networks and to derive well-being and self-esteem benefits (Morrison, 2002). Previous research suggests that people choose who they work with in the same way that they choose who they socialize with, based on liking over competence (Casciaro and Lobo, 2005). According to Casciaro and Lobo (2005), the reason for this is that when we like someone we feel that the resources they have are accessible to us and, therefore, that we can benefit from that relationship, whereas competence only implies presence of resources and not accessibility. As such, we expected that the same factors would drive popularity in both work and non-work networks.

### Method

#### Participants

Students from three psychology Masters courses at different UK universities were invited to participate in a study on how relationships develop; participation was not a course requirement. The first course comprised 27 students, 20 of whom provided data on all measurement occasions. The second comprised 18 students, 17 of whom completed all data points. The third course included 33 students, with full data from 31. The overall sample, therefore, comprised 68 participants (42 females and 24 males, *M*_age_ = 23.66 years, *SD* = 2.45), representing a response rate of 87%. Ethical approval for the study was obtained from the Department of Psychology Research Ethics Committee at the University of Sheffield in the UK (the institution where the first author formerly worked).

#### Design and Procedure

We used a longitudinal social network study design to assess whether use of IER predicted changes in participants’ popularity over time. Surveys were distributed during the students’ first semester of their courses (approximately a 12-week period). At baseline, 3 weeks into their course, students were given an introduction to the study and an opportunity to ask questions, and consented to take part in the research. They then provided a first measure of their work and non-work ties in their respective networks and completed measures of their demographic characteristics (gender and age) and personality (extraversion and agreeableness) and a scale assessing the extent of their use of IER toward their coursemates over the semester thus far. At the end of the semester, students completed a second measure of their work and non-work network ties.

#### Measures

##### Popularity

Participants’ popularity in the work and non-work networks was calculated on the basis of responses to two sociometric items, administered using a roster method. Participants were presented with a list of the people on their own Masters course, and asked to rate the extent (from 0 ‘not at all’ to 4 ‘a great extent’) to which they had shared specific types of relations (work and non-work) with each person during a defined time-period. In the Time 1 survey, participants rated the extent to which they had shared ties since they started the course; in the Time 3 survey, they rated the extent to which they had shared ties in the interim period since the first survey. For work ties, we asked participants to “please indicate the extent to which you have worked with each of your coursemates… By working together, we mean studying together at a library, collaborating on a course project, asking or giving advice on an academic topic – any university-related work activity.” For non-work ties, we asked participants to “please indicate the extent to which you have socialized with each of your coursemates outside of the University… By socializing, we mean going for a drink, going out for the night, going to the cinema, spending time in each others’ houses – any non-work leisure activity.”

Using responses to these items, we calculated participants’ *in-degree centrality* within their respective networks. As described earlier, in-degree centrality is a measure in social network analysis that indicates the extent to which others in a network have nominated a given network member (e.g., as someone they have worked or socialized with). It is often used as a measure of popularity in social network studies because the data is not self-reported by the network member in question, making it relatively objective (Sparrowe et al., 2001; Czarna et al., 2014). In this case, we calculated in-degree centrality (i.e., popularity) in the work and non-work networks. Finally, we divided the centrality values by network size, to control for differences between the networks (Scott and Judge, 2009; Czarna et al., 2014).

##### Interpersonal Emotion Regulation

Use of IER toward others in the networks was assessed using a self-report measure that has previously been validated against behavioral data (Niven et al., 2011). The measure was taken from the emotion regulation of others and self (EROS) scale, a comprehensive measure of emotion regulation that includes four subscales covering use of strategies to (i) improve one’s own feelings, (ii) worsen one’s own feelings, (iii) improve others’ feelings, and (iv) worsen others’ feelings. In this study, we used the subscale that assesses use of strategies to improve others’ feelings (termed ‘extrinsic affect-improving’). This subscale comprises six items (α = 0.88), with example items including: “I gave someone advice to try to improve how they felt” and “I made someone laugh to make them feel better”. Participants indicated the extent to which they had used these strategies toward their coursemates since the start of the semester (from 1 ‘not at all’ to 5 ‘a great deal’).

##### Personality Traits

Extraversion and agreeableness were each assessed using items each taken from the short version of the Big Five Inventory (Rammstedt and John, 2007). Participants indicated the extent to which they agreed (from 1 ‘disagree strongly’ to 5 ‘agree strongly’) with two items for extraversion (e.g., “I see myself as someone who is outgoing, sociable”; Spearman–Brown coefficient = 0.75) and two items for agreeableness (e.g., “I see myself as someone who is generally trusting”; Spearman–Brown coefficient = 0.67).

### Results

Means, standard deviations, and correlations between the main study variables are shown in **Table 1**. There was a high degree of overlap between popularity in the work and non-work networks: at baseline, *r* = 0.73, *p* < 0.01 (95% CIs 0.64, 0.85); and at end of semester, *r* = 0.82, *p* < 0.01 (95% CIs 0.78, 0.92). Correlations suggested that the use IER was significantly related to popularity at the end of the semester: in the work network, *r* = 0.40, *p* < 0.01 (95% CIs 0.14, 0.56); and in the non-work network, *r* = 0.29, *p* < 0.05 (95% CIs -0.01, 0.52). However, IER was not related to baseline popularity in the work, *r* = 0.19, *p* = 0.13 (95% CIs -0.09, 0.40) and non-work, *r* = 0.10, *p* = 0.40 (95% CIs -0.15, 0.36) networks, suggesting a lack of reverse causal relationship (i.e., that popularity is not associated with later use of IER).

**Table 1:** Correlations between main study variables in Study 1.

		Mean	*SD*	1	2	3	4	5	6	7	8
1	Age	23.66	2.45								
2	Gender	0.36	0.49	0.20							
3	Popularity in baseline work network	0.36	0.21	-0.04	-0.35^∗∗^						
4	Popularity in baseline non-work network	0.39	0.25	-0.22	-0.22	0.73^∗∗^					
5	Popularity in end of semester work network	0.48	0.32	-0.01	-0.37^∗∗^	0.69^∗∗^	0.53^∗∗^				
6	Popularity in end of semester non-work network	0.48	0.29	-0.11	-0.28^∗^	0.68^∗∗^	0.75^∗∗^	0.82^∗∗^			
7	Interpersonal emotion regulation (IER)	2.22	0.73	-0.31^∗^	-0.24	0.19	0.10	0.40^∗∗^	0.29^∗^		
8	Extraversion	3.37	1.00	0.04	-0.25^∗^	0.23	0.23	0.18	0.22	0.01	
9	Agreeableness	3.80	0.74	-0.13	-0.31^∗^	0.12	0.09	0.25^∗^	0.13	0.30^∗∗^	<0.01

N = 68; Gender was coded 0 for females, 1 for males. *p < 0.05, **p < 0.01.

Regression analyses were conducted to investigate whether the use of IER predicted a change in popularity across the semester. In these analyses, popularity at baseline, age, gender, extraversion, and agreeableness were controlled for. The results, shown in **Table 2**, indicate that IER strategies had a unique effect over personality in predicting change in popularity across the course of the semester, in both the work, β = 0.25, *p* < 0.01 (95% CIs 0.03, 0.19), and non-work, β = 0.21, *p* < 0.05 (95% CIs 0.01, 0.15), networks. The findings of this study, therefore, provide initial evidence that using IER toward others may be associated with relationship formation, in this case in face-to-face work and non-work networks.

**Table 2:** Regression analyses predicting change in social network popularity in Study 1.

	Centrality in work network at end of semester			Centrality in non-work network at end of semester		
	
	β	*t*	Δ*R*^2^	β	*t*	Δ*R*^2^
**Step 1**
Age	-0.02	-0.17		0.05	0.59	
Gender	-0.02	-0.22		-0.04	-0.40	
Centrality in work network at baseline	0.69	6.86^∗∗^				
Centrality in non-work network at baseline				0.77	8.48^∗∗^	
Extraversion	0.04	0.44		0.03	0.33	
Agreeableness	0.12	1.23	0.55^∗∗^	0.04	0.46	0.61^∗∗^
**Step 2**
Age	0.04	0.45		0.10	1.15	
Gender	<0.01	-0.02		-0.02	-0.18	
Centrality in work network at baseline	0.68	7.06^∗∗^				
Centrality in non-work network at baseline				0.77	8.78^∗∗^	
Extraversion	0.05	0.54		0.03	0.39	
Agreeableness	0.06	0.62		-0.01	-0.10	
IER	0.25	2.68^∗∗^	0.05^∗∗^	0.21	2.36^∗^	0.04^∗^
Total *R*^2^			0.60			0.65

*N = 68; Gender was coded 0 for females, 1 for males. ^∗^p < 0.05, ^∗∗^p < 0.01*.

## Study 2

In our second study, we wanted to determine whether the observed effects of IER on popularity could be replicated in online social networks. In other words, would the same psychological factors would be important in driving relationship formation online as face-to-face? We tested our central proposition using a dataset of Twitter users. Founded in 2006, Twitter is the world’s fastest growing online social networking site (ComScore, 2012), with 255 million monthly active users. Twitter allows users to post updates and messages, referred to as *tweets*, and to elect to subscribe to receive tweets from other users by *following* them. The number of followers a user has is, therefore, an indicator of a user’s popularity. The aim of the present study was to establish whether Twitter users’ engagement in IER via their tweets would predict their popularity. Drawing on data from a sample of over 8000 Twitter users from English-speaking countries, we used a linguistic tool to detect instances of IER in people’s *tweets* and tracked the activity of these users from the formation of their accounts.

A second aim of this study was to extend the findings reported in Study 1 by exploring whether cognitive and behavioral IER strategies would have different effects on popularity in this context. As discussed earlier, while behavioral IER ought to fulfill targets’ needs and so help to develop new relationships, cognitive IER could potentially be seen as a challenge to targets’ views and thus be taken as an offense. In online contexts, a difference between cognitive and behavioral IER may be particularly likely to be apparent, as written words that challenge a person may appear more abrasive due to the lack of non-verbal cues (Culnan and Markus, 1987).

### Method

#### Participants

Participants in the study were drawn from a dataset produced from a full sample of Twitter activity in 2013 that covers a large amount of Twitter users in different countries (Abisheva et al., 2013). Among these users, the participants selected for the present study were those from four English-speaking countries—USA, Canada, Australia, and the UK—who had at least one follower and at least one tweet mentioning another user by the designated point of analysis, and for whom we had access to almost all (over 95%) of the tweets they had generated. These criteria were important because we analyzed the content of tweets in English, were interested in interpersonal processes and so needed users who engaged at least somewhat with other members of Twitter, and wanted comprehensive documentation of users’ Twitter activity. The final sample comprised the 8605 Twitter users from the dataset who fulfilled these criteria, with up to 3200 tweets per user.

Although Twitter profiles do not have explicit information about demographics of users, meaning that we do not have demographic characteristics for the present sample, previous work has assessed the distributions of age, occupation, and gender of Twitter users. Twitter users in the US are somewhat more likely to be male, with 64% of users reported as male in 2013 (Garcia et al., 2014). The age distribution of Twitter users is clearly biased toward younger populations, but without very striking differences in occupation (Sloan et al., 2015).

Our analysis involved data voluntarily selected by participants to be publicly shared on Twitter. This public sharing explicitly includes third parties and thus provides clear consent to data access. In contrast with user interface manipulations that require careful ethical considerations, the present study does not control or manipulate the user interface and the analyses are performed over aggregations of users. Thus, following the principle of numerous previous studies on publicly available Twitter data (Golder and Macy, 2011; Mislove et al., 2011; Sloan et al., 2015), and consistent with principles of e-research ethics (Parker, 2010), no formal institutional ethics approval is required for this type of research.

#### Design and Procedure

We used a correlational study design in which we tracked each user from the database from the creation of their Twitter accounts starting with no followers to the point of analysis. This allowed us to determine whether the IER that users engaged in during their tweets predicted the development of new connections. The tweets used in the analysis were filtered, such that only tweets including an *@-mention* were selected. An @-mention in a tweet indicates that the person tweeting is communicating directly with another Twitter user. This is important because many tweets are not direct acts of communication with specific others (e.g., people may tweet general messages about a meal they just ate, or a place they have been to). In addition, we filtered out *re-tweets*, in which a user copies the content of another user, so that only original tweets were included in the analyses. Out of the total 10,170,651 tweets, our final pool included 4,250,112 tweets from the participants. We then coded each participant’s tweets to identify whether or not they represented an instance of IER (as described below).

#### Measures

##### Popularity

Popularity was measured as the number of followers users had gained since creating their accounts. Because people elect whether or not to follow a user, this is considered a suitable method of assessing popularity that is analogous to in-degree centrality. We applied a logarithmic transformation to the number of followers for our analysis. This type of transformation is commonly applied for data that are positively skewed (Quercia et al., 2012; Abisheva et al., 2013) and that follow power-law distributions (Clauset et al., 2009). In the present case, the skewness of the variable (pre-transformation) was 31.85. In our analyses on popularity, we also controlled for the age of the Twitter account, in recognition of the fact that people would have more time to gain followers with older accounts.

##### Cognitive and behavioral IER

Participants’ use of IER in their Twitter activity was inferred based on their use of particular terms in their tweets. Specifically, we coded all eligible tweets from participants using the dictionaries of the Linguistic Inquiry and Word Count (LIWC) tool (Pennebaker et al., 2007). LIWC is a software program that analyzes text for instances of particular words and terms to determine the extent to which different categories are used in that text.

We first coded all tweets for the presence of emotional terms, using the ‘affect’ category of the LIWC (which contains terms pertaining to positive and negative emotions). We then coded the tweets for presence of terms relating to the two main types of strategies to improve others’ emotions proposed in the dominant model of IER: (i) cognitive strategies, which involve trying to influence a person’s thoughts about his or her feelings or situation, e.g., giving someone advice; and (ii) behavioral strategies, which involve using behavior to change a person’s feelings, e.g., doing something nice for someone (Niven et al., 2009). To capture cognitive strategies, we coded the tweets for terms from the cognitive mechanisms category of the LIWC, which includes terms relating to logic, insight, causality, re-evaluation, thinking, and understanding. Such terms reflect the cognitive strategies included in Niven et al. (2009) classification of strategy types. Example tweets identified using this analysis as cognitive IER include “@XXX Since you have no control over your thoughts please don’t feel guilty about them...acting on them is a different matter” and “@XXX good plan. keep your head down and don’t answer any questions you’re asked. you should feel fine :)”. To capture behavioral IER, we coded for terms related to social processes in the LIWC. The expression of social process terms serves as a signal of social support (Tausczik and Pennebaker, 2010), including terms such as confiding, encouraging, flattering, giving, helping, and listening, which match well to the behavioral strategies in Niven et al. (2009) model. Example tweet identified as behavioral IER are “@XXX I’m sorry to hear that, Amy. Sending lots of hugs your way. Xo” and “@XXX most definitely. Can someone bring you a book and some distractions, perhaps? Would you like some cat sites I can send?”

Using this linguistic analysis, we then expressed each variable as a ratio, representing the number of tweets in which both cognitive and affect terms were used (for cognitive IER) or in which both social and affect terms were used (for behavioral IER) as a proportion of the total number of tweets sent by the user that fulfilled the filtering criteria outlined above (i.e., original tweets that included an @-mention). The resulting variables, therefore, represented the extent to which the user engaged in each type of IER in their Twitter activity.

### Results and Discussion

Descriptive statistics of the main study variables are shown in **Table 3**. The Twitter users produced an average of 111.39 tweets containing terms pertaining to cognitive IER (*SD* = 130.02), and an average of 127.19 tweets containing terms pertaining to behavioral IER (*SD* = 145.58), representing 22 and 26%, respectively, of all original interpersonal Twitter activity. There was a strong correlation between presence of terms connoting cognitive and behavioral IER in tweets, *r* = 0.76, *p* < 0.01 (95% CIs 0.75, 0.77). This overlap appeared to be due to the presence of emotion terms in both types of tweets, as additional analyses revealed that there was only a small correlation between presence of cognitive and behavioral terms in the tweets when emotion terms were held constant, *r* = 0.05, *p* < 0.01 (95% CIs 0.03, 0.07). Further analysis of the data revealed that 6% of tweets included in this study contained only cognitive IER (i.e., they did not also contain behavioral terms), while 9% of tweets contained only behavioral IER (i.e., they did not also contain cognitive terms).

**Table 3:** Correlations between main study variables in Study 2.

	Mean	*SD*	1	2	3	4
1. Age of the account (days)	842.69	523.11				
2. Number of tweets	493.91	497.80	0.10^∗∗^			
3. Number of followers	463.82	3751.05	0.20^∗∗^	0.21^∗∗^		
4. Use of cognitive IER terms in tweets	0.22	0.10	0.04^∗∗^	0.06^∗∗^	-0.02^∗^	
5. Use of behavioral IER terms in tweets	0.26	0.13	0.03^∗∗^	-0.04^∗∗^	0.12^∗∗^	0.76^∗∗^

*N = 8605; Number of followers is analyzed in raw form for mean and SD, and as a logarithmic transform for correlations ^∗^p < 0.05, ^∗∗^p <0.01*.

Despite the overlap between cognitive and behavioral IER, both appeared to have distinct relations with popularity. The use of tweets connoting cognitive IER had a small negative relationship with users’ popularity, *r* = -0.02, *p* < 0.05 (95% CIs -0.04, -0.001), whereas use of behavioral IER in tweets was positively related to popularity, *r* = 0.12, *p* < 0.01 (95% CIs 0.10, 0.14). Among the tweets that included only cognitive or only behavioral IER, there were stronger correlations with popularity in the same direction as those reported above (cognitive IER, *r* = -0.18, *p* < 0.01; behavioral IER, *r* = 0.16, *p* < 0.01).

A regression analysis was then conducted, in which the age of the Twitter account and the total number of tweets users had sent out were controlled for in Step 1 (to account for differences in time to gain followers and usage of Twitter), and both types of IER were entered as predictors in Step 2 (**Table 4**). The results at Step 2 indicated that use of behavioral IER in one’s tweets was positively related to network popularity, β = 0.49, *p* < 0.01 (95% CIs 0.45, 0.53), while cognitive IER was negatively related to popularity, β = -0.44, *p* < 0.01 (95% CIs -0.48, -0.39). At Step 3 the interaction between cognitive and behavioral IER was added to the model to determine whether presence of both cognitive and behavioral terms in tweets would have additional predictive value in terms of popularity. The interaction was not significant, β < 0.01, ns (95% CIs -0.01, 0.02). The findings of this study, therefore, replicate the first study in suggesting that IER may be associated with the development of new connections with others, but present a more nuanced picture, at least for online connections, in suggesting that only behavioral strategies may be positively related to popularity.

**Table 4:** Regression analysis predicting Twitter popularity in Study 2.

	Number of followers		
	β	*t*	Δ*R*^2^
**Step 1**
Age of the account (days)	0.17	11.43^∗∗^	
Number of tweets	0.55	36.26^∗∗^	0.17^∗∗^
**Step 2**
Age of the account (days)	0.18	11.94^∗∗^	
Number of tweets	0.56	37.42^∗∗^	
Use of cognitive IER terms in tweets	-0.44	-19.68^∗∗^	
Use of behavioral IER terms in tweets	0.49	22.06^∗∗^	0.05^∗∗^
**Step 3**
Age of the account (days)	0.18	11.95^∗∗^	
Number of tweets	0.56	37.34^∗∗^	
Use of cognitive IER terms in tweets	-0.44	-19.22^∗∗^	
Use of behavioral IER terms in tweets	0.49	22.05^∗∗^	
Interaction: cognitive × behavioral IER	<0.01	0.51	<0.01
Total *R*^2^			0.21

*N = 8605; ^∗^p < 0.05, ^∗∗^p < 0.01*.

## General Discussion

How do we make connections when we enter a new social situation? The present research suggests that making attempts to improve others’ emotions may facilitate the formation of new relationships. Across two studies, we found that use of IER was associated with attraction of new network connections, in face-to-face and online contexts, and in work and non-work relationships. IER may, therefore, have an important role to play in helping people to become popular.

Our research suggested that the effects of IER may not always be positive when it comes to popularity, however. In our second study, we contrasted two types of strategies for IER. We found that while use of terms relating to behavioral IER in tweets was associated with higher popularity in terms of Twitter followers gained, use of terms relating to cognitive IER was associated with lower popularity. The negative associations between cognitive IER and popularity observed in the present research stand in contrast to research on emotion self-regulation, in which cognitive strategies, such as reappraisal, are generally found to have positive consequences for both affect and social relations (Gross and John, 2003). A possible issue with cognitive strategies when it comes to regulating others’ emotions is that even though they are used with the intention of improving the target’s affect, they could be construed as a challenge to the target’s views and thus taken as an offense (e.g., a person who is upset about criticism from his manager could find a colleague’s suggestion that the manager is only trying to improve his performance insensitive to his feelings or as taking the manager’s side). Although this unintended impact may not always transpire, it may be especially likely during CMCs where the lack of non-verbal cues may mean that any confusion over someone’s intentions is difficult to resolve and offense may be taken more quickly (Culnan and Markus, 1987). Thus, consistent with research suggesting that attempts to provide social support that are not perceived to be responsive to the intended target’s needs may backfire (Maisel and Gable, 2009), cognitive IER may also fail to achieve relational benefits, at least in online communications. An alternative explanation for the negative association between cognitive IER and popularity found in our second study; however, is that there may have been issues with the coding of cognitive strategies (discussed later in more detail), such that tweets that did not include cognitive IER may have been included in the analysis.

The present research makes three key contributions to the literature. First, it makes a broad contribution to the field of the social nature of emotions. Research on emotion regulation has paralleled that in the field of emotion, in that the *social nature* of this process has been recognized more widely in recent years. For example, studies have reported that people’s regulation of their emotions is often engaged during or in anticipation of social interactions (Erber et al., 1996) and that people can recruit the aid of others in regulating their feelings (Fitzsimons and Finkel, 2010). One of the most important advances in this area is the recognition that as well as regulating their own emotions, people can also intentionally try to shape the way others feel (i.e., they can engage in IER), yet to date empirical research on this process has been somewhat sparse. In the present paper, we not only demonstrate the everyday use of this social process of IER in both face-to-face and online relationships, but we also explicitly connect it to its social consequences, by showing that it can have implications for relationship formation.

Second, the research makes a more specific contribution toward our understanding of the differential effects of distinct types of IER. Despite two main types of strategies to improve others’ affect being proposed in the dominant model of IER (Niven et al., 2009), to-date most studies have only contrasted strategies to improve and to worsen affect. The present research theorizes that each type of strategy may have differential effects due to the way in which the strategy is likely to be appraised and presents the first clear evidence that cognitive and behavioral strategies have different effects when used in real relationships. Behavioral strategies communicate support, comfort, and validation, and so are likely to be positively appraised and facilitate the formation of new relationships over time. In contrast, cognitive strategies may be perceived as a challenge to the target’s way of viewing a situation, and so may not always aid in building new relationships. Our findings in this respect are in line with earlier work on different listening styles (Nils and Rimé, 2012), but extend this work by studying the spontaneous use of IER strategies in the naturalistic context of newly developing relationships. However, it should of course be noted that we only tested and observed differences between cognitive and behavioral IER within online relationships. Future studies should, therefore, compare the effects of these strategy types in face-to-face social networks.

Third, our research contributes by extending the theoretical understanding of how social networks develop over time. The importance of building informal network ties, especially for newcomers (e.g., in work organizations) is well-established (Morrison, 2002), yet there has been a relative dearth of research examining psychological factors—especially those that are within a person’s control—that predict formation of new ties (Totterdell et al., 2008). The present research suggests that IER may potentially be an important process in facilitating popularity in new networks, even when personality traits that have previously been thought to be important determinants of popularity (i.e., extraversion and agreeableness) are taken into account. Our research, therefore, highlights the central nature of pleasant feelings to relationship formation. Specifically, because IER can elicit positive affect (Niven et al., 2007), people may wish to repeat exchanges with IER users in order to experience more of these rewarding feelings (Lawler, 2001) or to share the effort involved in emotion regulation with the interaction partner (Beckes and Coan, 2011). On a practical level, our findings offer a key set of strategies that people may be able to engage in to facilitate the formation of relationships when entering a new social context. They further highlight that similar types of strategies can help to develop online and face-to-face relations, and work and non-work relations (consistent with previous research on factors driving work partner choices; Casciaro and Lobo, 2005).

The present research has several strengths, notably: the use of relatively objective indices of popularity; the study of real emerging relationships over time; and that we explored the effects of IER on the formation of three different types of relationships. However, certain limitations of the research must be acknowledged. In particular, an issue across both studies is that we used aggregate measures to assess popularity. Although this provides the most accurate way of capturing the views of a whole network of people, a potential problem is that our findings only tell us about networks on average, and not about the fate of particular relationships. As such, while using IER might help people to form more relationships overall, it could still, in theory, cause some relationships to be cut off. In addition, the correlational design of our studies also means that evidence for causality is not unequivocal. By studying networks from their formation with measures separated temporally across a 3-month period (Study 1), and studying Twitter users from the point at which they started their accounts (Study 2), we overcame some of the typical issues associated with correlational study design. Moreover, while it is still possible based on our study design that popularity might cause the use of IER as well as the reverse, the findings of Study 1 suggest this was unlikely to be the case, given that baseline popularity did not predict later use of IER. Nevertheless, the use of experimental study designs to establish causality more directly will be important to complement the findings we have observed here.

A final limitation specifically in reference to Study 2 concerns the indirect nature of our measures of IER. In this study, we were only able to infer the use of IER by using linguistic analysis of people’s tweets to other users. Analyzing the content of online communication for social sharing of emotions (Garas et al., 2012) and emotion-related processes (e.g., empathy; Pfeil and Zaphiris, 2007) is an established means of studying relationship formation, and the tool we applied is widely used and robust (Garcia et al., 2012). However, IER is defined in terms of the intention of the regulator to affect a change in someone else’s feelings (Niven et al., 2009), and intentionality cannot be fully captured without directly questioning the regulator. Moreover, there are possible instances of IER that may not have been picked up using our coding system (e.g., if someone were to use IER without explicitly referring to an emotion term) as well as possible instances where tweets could have been coded as IER erroneously. Likewise, there could be potential for miscoding of cognitive strategies as behavioral and vice versa, due to the overlap in terms likely to relate to each strategy type (e.g., the term ‘understanding’ was featured in the category used to code for cognitive IER, even though the notion of behavioral IER concerns communication of understanding to the target). Future research examining use of IER in online communications should, therefore, consider cross-validating coding, for example, by correlating IER as inferred from online social network messages with self-reported use of IER as indicated on an established measure, such as the EROS scale (Niven et al., 2011).

Future research should also explore whether our findings translate to other social contexts. The fact that we found similar patterns of results regarding different types of relationships, and that in Study 1 we included three different networks of people (i.e., three Masters courses) and found the same patterns of results within each (exploratory moderation analyses examining differences between the networks in Study 1 revealed no significant variations), is encouraging. However, additional research conducted with other samples of people entering new social contexts (e.g., people starting new jobs, moving to new neighborhoods, or joining new leisure clubs) would provide further confidence in the generalizability of our findings.

Another direction for future research will be to consider situations under which IER does not lead to expected gains in popularity. One possible factor to consider here will be motives for IER. Recent research has highlighted that people do not always have others’ interests in mind when engaging in IER. Specifically, across a series of studies, Netzer et al. (2015) demonstrated that people may regulate others’ emotions in order to pursue personal instrumental goals. While in the present research, we have reported evidence that trying to improve others’ emotions is associated with formation of new relationships; future research could study whether people’s motivations for using IER (or others’ perceptions of their motives) will influence how successful IER is in boosting popularity.

## Author Contributions

KN led the design, data collection, and analysis of Study 1, contributed to the design of Study 2, and produced the first and revised drafts of the paper. DG led the design and analysis of Study 2 and helped to revise the paper. IL, DH, and WM contributed to the design and data collection of Study 1 and helped to revise the paper. All authors are accountable for the accuracy or integrity of the work.

## Conflict of Interest Statement

The authors declare that the research was conducted in the absence of any commercial or financial relationships that could be construed as a potential conflict of interest.
